# *Anaplasma phagocytophilum*-Occupied Vacuole Interactions with the Host Cell Cytoskeleton

**DOI:** 10.3390/vetsci3030025

**Published:** 2016-09-21

**Authors:** Hilary K. Truchan, Chelsea L. Cockburn, Levi J. May, Lauren VieBrock, Jason A. Carlyon

**Affiliations:** Department of Microbiology and Immunology, School of Medicine, Virginia Commonwealth University Medical Center, Richmond, VA 23298, USA; hilary.truchan@northwestern.edu (H.K.T.); cockburnc@mymail.vcu.edu (C.L.C.); maylj2@mymail.vcu.edu (L.J.M.); lviebrock@gmail.com (L.V.)

**Keywords:** *Anaplasma phagocytophilum*, vacuole-adapted bacteria, vimentin, SUMOylation, cytoskeleton

## Abstract

*Anaplasma phagocytophilum* is an obligate intracellular bacterial pathogen of humans and animals. The *A. phagocytophium*-occupied vacuole (ApV) is a critical host-pathogen interface. Here, we report that the intermediate filaments, keratin and vimentin, assemble on the ApV early and remain associated with the ApV throughout infection. Microtubules localize to the ApV to a lesser extent. Vimentin, keratin-8, and keratin-18 but not tubulin expression is upregulated in *A. phagocytophilum* infected cells. SUMO-2/3 but not SUMO-1 colocalizes with vimentin filaments that surround ApVs. PolySUMOylation of vimentin by SUMO-2/3 but not SUMO-1 decreases vimentin solubility. Consistent with this, more vimentin exists in an insoluble state in *A. phagocytophilum* infected cells than in uninfected cells. Knocking down the SUMO-conjugating enzyme, Ubc9, abrogates vimentin assembly at the ApV but has no effect on the bacterial load. Bacterial protein synthesis is dispensable for maintaining vimentin and SUMO-2/3 at the ApV. Withaferin A, which inhibits soluble vimentin, reduces vimentin recruitment to the ApV, optimal ApV formation, and the bacterial load when administered prior to infection but is ineffective once vimentin has assembled on the ApV. Thus, *A. phagocytophilum* modulates cytoskeletal component expression and co-opts polySUMOylated vimentin to aid construction of its vacuolar niche and promote optimal survival.

## 1. Introduction

*Anaplasma phagocytophilum* is a tick-transmitted bacterium that for almost 60 years was considered as solely a veterinary pathogen until 1994, when it was identified as the etiologic agent of a febrile illness that afflicted several human patients in Minnesota and Wisconsin [1,2,3]. In all, *A. phagocytophilum* infects humans and a variety of wild and domestic animal species. Fatal infections have thus far been reported in sheep, cattle, horses, reindeer, roe deer, moose, dogs, and humans [3]. As infection is accompanied by granulocytic cytoplasmic bacterial inclusions, the disease was ultimately ascribed the term, granulocytic anaplasmosis [4,5,6]. Human granulocytic anaplasmosis (HGA) is an acute illness accompanied by non-specific symptoms including fever, chills, myalgia, headache, leukopenia, thrombocytopenia, and elevated liver enzymes [4,7]. HGA can be more serious or fatal in immunocompromised or elderly patients and when antibiotic therapy is delayed [5,8], with 36% of symptomatic patients requiring hospitalization and 7% requiring intensive care [9]. The number of cases of HGA reported annually to the United States Centers for Disease Control rose nearly seven-fold from 2002 to 2012, the last year for which statistics are available [8], though the disease remains largely underreported [3].

*A. phagocytophilum* infects granulocytes and endothelial cells to replicate in a host-derived vacuole termed the *A. phagocytophilum*-occupied vacuole (ApV). In the laboratory, human promyelocytic HL-60 cells and primate RF/6A endothelial cells are useful models for studying *A. phagocytophilum* infection because they express receptors that the bacterium utilizes for invasion [10,11,12,13]. Additionally, RF/6A cells are particularly useful for examining the cellular microbiology of *A. phagocytophilum* infection because they are large, adherent, and flat, which makes them ideal for imaging [11,14,15,16,17]. The ApV remains intact throughout the infection cycle and expands to accommodate the growing number of bacteria. ApV expansion is likely linked, at least in part, to the acquisition of membranes from autophagosomes [18], *trans*-Golgi vesicles [15], and ER-derived vesicles [16] that are directed to the ApV. As these and other vesicles are anchored to and transported by the cytoskeleton, it is likely that the ApV interacts with cytoskeletal components. Additionally, because the ApV forms at the plasma membrane and migrates to the perinuclear space [15], it might hijack cytoskeletal components for motility. ApV-cytoskeletal component interactions, however, are insufficiently defined.

The three main structural components of the cytoskeleton are microtubules, microfilaments and a group of polymers known as intermediate filaments. Microtubules are polymers of heterodimers of α and β tubulin while microfilaments are actin homopolymers [19]. Intermediate filaments are comprised of a diverse family of more than 70 proteins that assemble either as heteropolymers, examples of which are Type I and II keratins, or homopolymers, an example of which is vimentin [20]. Both actin filaments and microtubules are polarized polymers that depolymerize and repolymerize to contribute to cell migration and division and interface with molecular motors to regulate intracellular vesicle trafficking [21]. All three components contribute to the structural stability and mechanical support of eukaryotic cells. Intermediate filaments assemble to combat different cellular stresses, help to anchor and support organelles within the cell, and recent evidence suggests that they also regulate protein and vesicular trafficking [21,22,23,24]. Whether the ApV and host cell cytoskeleton interact is poorly understood. Like many intracellular pathogens [25], *A. phagocytophilum* entry into its host cell is at least partially dependent on actin [26]. Once inside the cell, the bacterium also targets vimentin to the ApV to modulate extracellular signal-related kinases 1 and 2 (Erk1/2) signaling and promote infection [17]. Further studies on vimentin and potential involvement of the other cytoskeletal components during infection have not been explored.

SUMOylation, the process by which small ubiquitin-like modifiers (SUMO) are covalently attached to proteins in a readily reversible process by a series of SUMO-specific enzymes, is an essential posttranslational modification in eukaryotes. Conjugation of SUMO moieties (SUMO-1, SUMO-2, SUMO-3) involves the E2 ubiquitin ligase ubiquitin-conjugating enzyme 9 (Ubc9), which targets lysine residues within a consensus motif for modification [27,28]. SUMO-1 is mostly contained within the nucleus and conjugated as a monomer, whereas SUMO-2 and SUMO-3, which are nearly identical in sequence, are contained within the nucleus and cytosol and can be conjugated as polymers [29]. SUMO-1 also terminates SUMO-2/3 polymers [30]. SUMOylation can result in one or more of three possible effects. First, the SUMO moiety may function as an interface for new interacting protein partners or conversely, block existing protein interactions. Second, the SUMO modification may alter the localization of the protein within the cell. Third, the modification may cause a conformational change in the protein that directly affects its stability and activity [14,28,31]. Intermediate filaments can be SUMOylated, a modification that regulates filament formation and solubility. Keratins and vimentin are extensively and preferentially modified by SUMO-2 and SUMO-3 but not SUMO-1 in vitro [29,32], which is consistent with keratin and vimentin being cytoplasmic proteins and SUMO-1 being predominantly found within the nucleus. Vimentin SUMOylation has only been detected in vitro, whereas keratin SUMOylation has been more extensively studied and confirmed to occur in vivo. Keratin is not SUMOylated under basal conditions but is extensively SUMOylated under conditions of cellular stress, including injury and oxidative and apoptotic stress [29,32]. SUMOylation of intermediate filaments during the course of intracellular bacterial infection has not been described.

In this study, we report that keratin and vimentin extensively assemble on the ApV. Microtubules also assemble on the ApV, but to a much lesser degree. Vimentin and SUMO-2/3 heavily label and colocalize at the ApV throughout infection. This suggests that vimentin might be hyperSUMOylated during *A. phagocytophilum* infection, which could influence its association with the ApV. Indeed, knockdown of Ubc9 results in a loss of vimentin assembly at the ApV. Bacterial protein synthesis is not needed to maintain vimentin and SUMO-2/3 moieties at the ApV. Likewise, once vimentin has been recruited to surround the ApV, it is insensitive to withaferin A (WFA). However, pretreatment with WFA inhibits vimentin recruitment to the ApV, optimal ApV formation, and reduces the bacterial load. These results advance understanding of interfaces that occur between the pathogen-occupied vacuole, host cytoskeleton, and posttranslational processes during intracellular *A. phagocytophilum* infection.

## 2. Materials and Methods

### 2.1. Cultivation of Uninfected and A. phagocytophilum Infected Host Cell Lines

Uninfected and *A. phagocytophilum* (NCH-1 strain) infected human promyelocytic HL-60 cells, CCL-240; American Type Culture Collections (ATCC, Manassas, VA, USA), RF/6A rhesus monkey choroidal endothelial cells (CRL-1780; ATCC) and HEK-293T cells were cultured as described previously [15,33].

### 2.2. Antibodies

Commercial primary antibodies used were: mouse anti-keratin-8 (Sigma-Aldrich, St. Louis, MO, USA), mouse anti-keratin-18 (Sigma-Aldrich), mouse anti-vimentin (Abcam, Cambridge, MA, USA), rabbit anti-SUMO-1 (Abcam), rabbit anti-SUMO-2/3 (Abcam), rabbit anti-Ubc9 (Santa Cruz Biotechnology, Dallas, TX, USA), mouse anti-β-actin (Santa Cruz Biotechnology), and mouse anti-human GAPDH (Sigma-Aldrich). Mouse and rabbit antisera against APH0032 were described previously [33]. Rabbit antiserum against *A. phagocytophilum* protein P44 was described previously [34]. Mouse anti-β-tubulin and Alexa Fluor phalloidin 555 were kind gifts from Jessica Bell (University of San Diego, San Diego, CA, USA) and Lynne Elmore (Virginia Commonwealth University, Richmond, VA, USA), respectively. Secondary antibodies conjugated to Alexa Fluor fluorochromes were obtained from Thermo Fisher Scientific (Waltham, MA, USA).

### 2.3. Immunofluorescence Assays and Microscopy

Cells for immunofluorescence assays were grown and infected on 12-mm glass coverslips (Electron Microscopy Sciences, Hatfield, PA, USA). The cells were fixed in 4% paraformaldehyde (Electron Microscopy Sciences) for 20 min at room temperature followed by permeabilization with 0.5% Triton X-100 for 10 min and screening with antibodies for immunofluorescence microscopy as described previously [35]. Coverslips were mounted with Prolong Gold Anti-fade reagent with DAPI (4’,6-diamidino-2-phenylindole, Thermo Fisher Scientific). Images were obtained using a Zeiss LSM 700 laser-scanning confocal microscope (Zeiss, Oberkochen, Germany). LSCM was performed at the VCU Department of Anatomy and Neurobiology Microscopy Facility, which is supported, in part, by funding from NIH-NINDS Center core grant (5P30NS047463).

### 2.4. Infection Assays

HL-60 cells were infected with *A. phagocytophilum* organisms released from infected HL-60 cells by sonication as previously described [36]. RF/6A or HEK-293T cells were infected with *A. phagocytophilum* bacteria that had been naturally released from infected RF/6A cells into the culture media as previously described [15]. In some cases, infected RF/6A cells on 12-mm glass coverslips were treated at 24 h post infection with 10 µg/mL oxytetracycline hydrochloride (Sigma-Aldrich) in 70% ethanol or vehicle control (70% ethanol) for up to 5 h. After treatment, the cells were washed with phosphate-buffered saline (PBS), the culture media was replaced, and the cells were processed for LSCM analyses.

### 2.5. Western Blot Analyses

Cell lysates were analyzed by SDS-PAGE and Western blot as previously described [37] with the exception that the insoluble fraction was solubilized in SDS sample buffer and combined with the detergent soluble fraction when analyzing whole cell lysates. The detergent solubility assay was performed as previously described [20]. In brief, uninfected and infected cells were lysed in TNEX buffer (50 mM Tris-HCl, pH 7.4, 150 mM NaCl, 5 mM EDTA, and 0.1% Triton X-100) and centrifuged to form the detergent-soluble supernatant and detergent-insoluble pellet. The insoluble pellet was washed with TNEX and solubilized in SDS sample buffer.

### 2.6. qRT-PCR

Total RNA isolated from uninfected and *A. phagocytophilum* infected host cells was analyzed by qRT-PCR as previously described [37,38]. Relative *vimentin* transcript levels were normalized to *β-actin* (please confirm whether this should be in italic) transcript levels using the 2^−ΔΔCT^ method [39]. Primers targeting human vimentin (5′-AGGAATGGCTCGTCACCTTCGTGAATA-3′ and 5′-GGAGTGTCGGTTGTTAA GAACTAGAGCT-3′) [40] were kindly provided by Zendra Zehner (Virginia Commonwealth University, Richmond, VA, USA). Primers targeting human actin were 5′-AGAGGGAAATCGTGCGTGAC-3′ and 5′-CAATAGTGATGACCTGGCCGT-3′ [14].

### 2.7. UBC9 siRNA Knockdown

4 × 10^5^ HEK-293T cells were seeded onto 12-mm glass coverslips (Electron Microscopy Sciences). After 16 to 20 h, 80 µL of 5 µM ON-TARGETplus human UBC9 siRNA SMARTpool (GE Dharmacon, Lafayette, CO, USA) was mixed with 320 µL of media and added to the wells. Non-targeting or GAPDH targeting siRNA (GE Dharmacon) was added to control wells. After 72 h, 200 µL of media containing *A. phagocytophilum* organisms that had been naturally released from infected RF/6A cells. Additionally, cells from one well of each siRNA treatment were harvested for Western blot analysis to confirm knockdown. At 48 h post-infection, cells were processed for microscopy analyses.

### 2.8. Assessment of the Effect of WFA on A. phagocytophilum Infection

RF/6A cells on 12-mm glass coverslips were pretreated with 0.5 or 1 µM WFA (Sigma Aldrich) for 2 h and incubated in 200 µL of media containing *A. phagocytophilum* organisms that had been naturally released from infected RF/6A cells. At 24 h, the cells were processed for LSCM. To determine the effect of WFA on the A. phagocytophilum DNA load, a similar experiment was performed except that RF/6A cells seeded in a 25 cm^2^ flask were infected with 1 mL of media containing *A. phagocytophilum* organisms prior to DNA isolation and QPCR as previously described using primers targeting Ap 16s rDNA and host cell β-actin. Relative Ap 16s rDNA levels were normalized to β-actin levels using the 2^−ΔΔCT^ method [39]. In some instances, *A. phagocytophilum* infected RF/6A cells were treated with 40 µM WFA for 3 h at 24 h post-infection prior to LSCM and QPCR.

### 2.9. Statistical Analyses

Student’s *t*-test was performed using the Prism 5.0 software package (Graphpad, San Diego, CA, USA) to assess statistical significance. Statistical significance was set at *p* ≤ 0.05.

## 3. Results

### 3.1. Intermediate Filaments and Microtubules Assemble around the ApV

**Figure 1:**
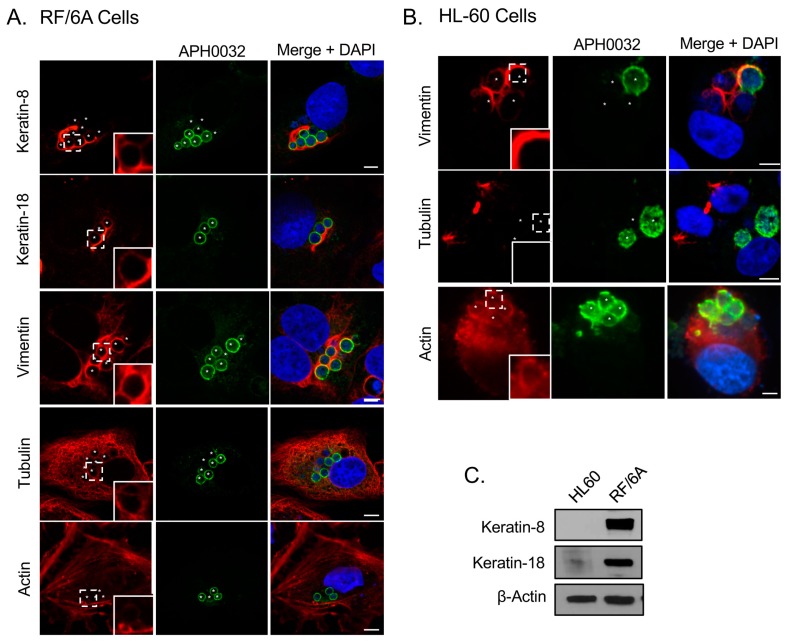
Intermediate filaments and microtubules assemble around the ApV. (**A**) Keratin-8, keratin-18, tubulin, and vimentin assemble at the ApV in RF/6A cells. *A. phagocytophilum* infected RF/6A cells that had been labeled with APH0032 and keratin-8, keratin-18, tubulin, or vimentin antibodies or rhodamine-phalloidin probe were visualized using laser-scanning confocal microscopy (LSCM); (**B**) Vimentin assembles at the ApV in HL-60 cells. *A. phagocytophilum*-infected HL-60 cells that had been labeled with APH0032 and vimentin, tubulin, or actin antibodies were visualized using LSCM; (**C**) Keratin-8 and keratin-18 are not expressed in HL-60 cells. Lysates of uninfected HL-60 and RF/6A cells were analyzed by Western blot with keratin-8, keratin-18, and β-actin antibodies. (**A**,**B**) Regions that are demarcated by hatched lined boxes correspond to the regions magnified in the insets that are demarcated by solid lined boxes. Asterisks (*) denote ApVs. Host nuclei and bacterial DNA were stained with DAPI (blue). Scale bars, 5 μm. Results in all panels are representative of two to three experiments with similar results.

**Figure 2:**
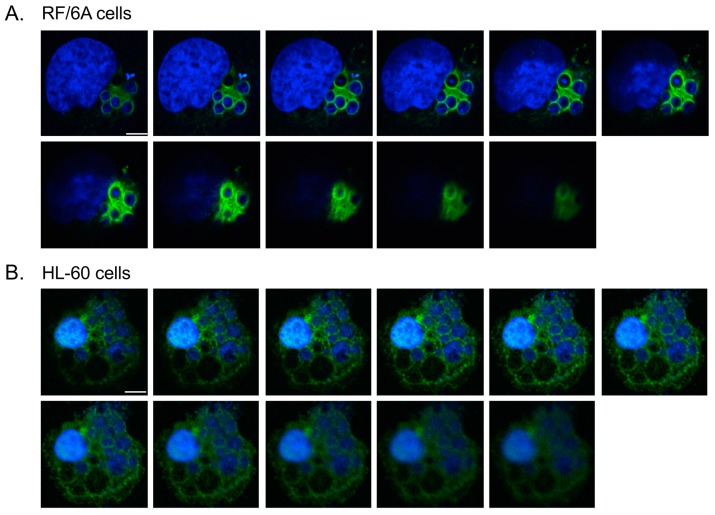
Z-stack analyses confirm vimentin assembly around the ApV. *A. phagocytophilum* infected RF/6A (**A**) and HL60 (**B**) cells were labeled with vimentin antibody and stained with DAPI. LSCM and Z-stack analyses were performed. Scale bars, 5 μm. Results in panels are representative of three experiments with similar results.

To determine if the ApV interacts with cytoskeletal components, localization of markers for microtubules (β-tubulin), microfilaments (actin), and intermediate filaments (keratin-8, keratin-18, and vimentin) was surveyed in infected RF/6A cells by confocal microscopy. Keratin-8 and keratin-18 are keratin monomers that pair together to form dimers and eventually filaments. These monomers are found in epithelial cells [32,41]. They and vimentin are, however, aberrantly expressed in cancer cells [42,43] and are thus sometimes expressed in non-epithelial immortalized cell lines [44]. β-tubulin, keratin-8, keratin-18 and vimentin were visualized with antibodies while filamentous actin was visualized with a fluorescent derivative of phalloidin. To visualize the ApV, antisera against APH0032, an *A. phagocytophilum* protein that localizes to the ApV membrane late (~24 to 28 h) in the infection cycle [33], was used. Given that infection in the cells had become asynchronous when they were examined, APH0032 would be absent on less mature ApVs and thus not all ApVs would be denoted by APH0032 antibody. Therefore, DAPI, which labels both bacterial and host DNA, was used to denote intravacuolar *A. phagocytophilum* organisms as well as host cell nuclei, respectively. Keratin-8 and keratin-18 expression in RF/6A endothelial cells was confirmed using confocal microscopy and Western blot (Figure 1A,C). Keratin-8 and keratin-18 formed robust rings around ApVs (Figure 1A). In agreement with a previous report [17], vimentin was also observed to be pronouncedly reorganized around ApVs. In contrast to intermediate filament components, tubulin faintly localized to the ApV and association of filamentous actin with the ApV could not be detected (Figure 1A). These results suggest that the ApV interacts with two cytoskeletal components, intermediate filaments and microtubules. To confirm these results in another host cell type, these analyses were extended to *A. phagocytophilum* infected HL-60 cells. Actin, but neither keratin-8 nor keratin-18 was detected in whole lysates of uninfected HL-60 cells by Western blot (Figure 1C). Vimentin was detected surrounding ApVs, but tubulin was not (Figure 1B). Actin was detected around the periphery of ApVs. Yet, given the abundance of actin in the host cells, it could not be definitively concluded whether actin was specifically recruited to ApVs. Z-stack analyses confirmed that vimentin accumulated around ApVs in both RF/6A and HL-60 cells in all focal planes examined (Figure 2). These results demonstrate that the intermediate filament vimentin robustly associates with ApVs in both promyelocytic and endothelial cells.

### 3.2. SUMO-2/3 but Not SUMO-1 Colocalizes with Vimentin Assembled around the ApV

**Figure 3:**
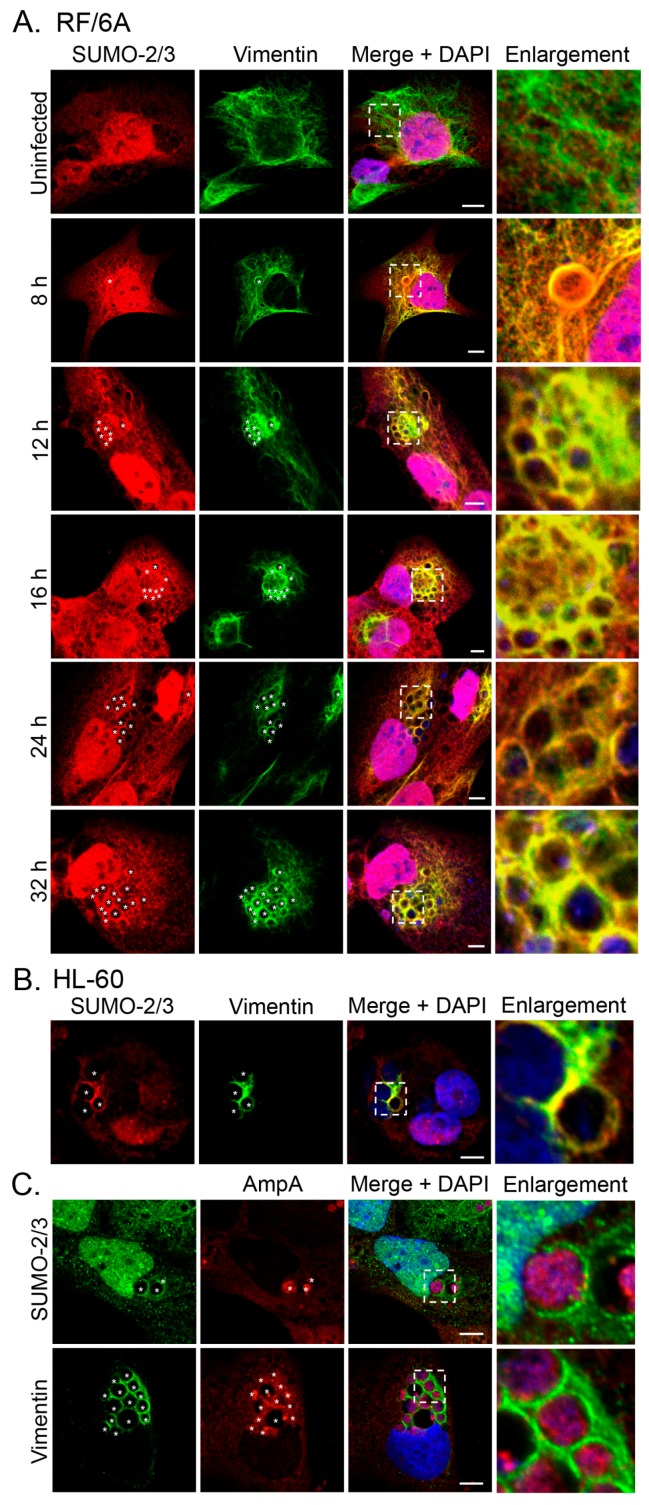
SUMO-2/3 colocalizes with vimentin assembled around the ApV. (**A**) Vimentin and SUMO-2/3 strongly colocalize in infected RF/6A cells. RF/6A cells that had been infected with *A. phagocytophilum* for 8, 12, 16, 24, or 32 h and uninfected cells were labeled with SUMO-2/3 and vimentin antibodies and examined by LSCM; (**B**) Vimentin and SUMO-2/3 strongly colocalize in infected HL-60 cells. *A. phagocytophilum*-infected HL-60 cells were labeled with SUMO-2/3 and vimentin antibodies and examined by LSCM; (**C**) The localization pattern of SUMO-2/3 and vimentin around the ApV extend beyond the pattern of the known SUMOylated *A. phagocytophilum* protein, AmpA, that localizes to the ApV membrane. *A. phagocytophilum*-infected RF/6A cells were labeled with AmpA and vimentin or SUMO-2/3 antibodies and examined by LSCM. (**A**–**C**) Asterisks (*) denote ApVs. Host nuclei and bacterial DNA were stained with DAPI (blue). Scale bars, 5 μm. Regions that are demarcated by hatched lined boxes correspond to the regions magnified in the enlargement. Results in all panels are representative of two to three experiments with similar results.

**Figure 4:**
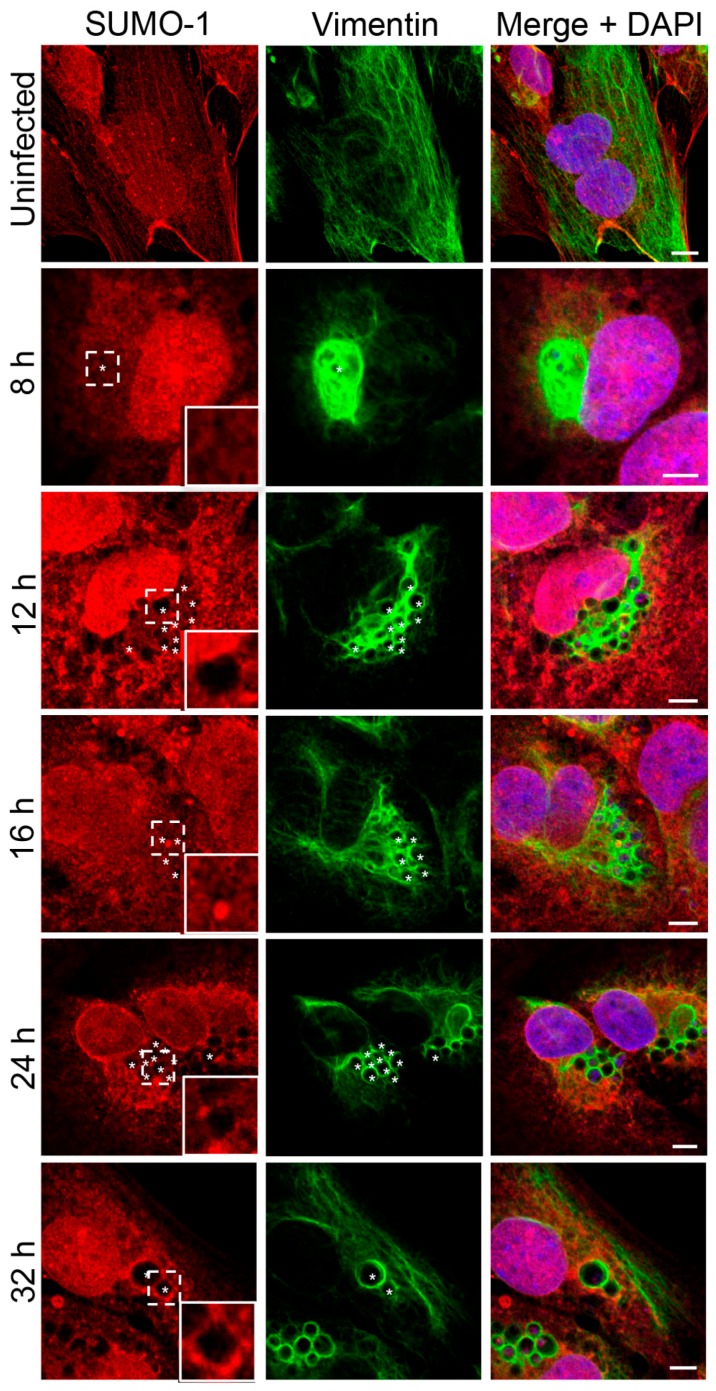
SUMO-1 does not colocalize with vimentin assembled around the ApV. RF/6A cells that had been infected with *A. phagocytophilum* for 8, 12, 16, 24, or 32 h and uninfected cells were labeled with SUMO-1 and vimentin antibodies and examined by LSCM. Regions that are demarcated by hatched lined boxes correspond to the regions magnified in the insets that are demarcated by solid lined boxes. Asterisks (*) denote ApVs. Host nuclei and bacterial DNA were stained with DAPI (blue). Scale bars, 5 μm. Results in all panels are representative of two experiments with similar results.

In our previous study of the A. phagocytophilum effector AmpA that becomes directly SUMOylated during infection of host cells [14], we made the fortuitous observation that SUMO-2/3 moieties pronouncedly localize around the ApV in a filamentous pattern. SUMOylation of intermediate filaments during formation and under various cellular stresses, though not bacterial infection, has been previously described [29,32]. Given that vimentin and keratin filaments assemble at the ApV, we reasoned that vimentin and/or keratin might be modified by SUMO-2/3. As a first step in testing this hypothesis, RF/6A cells synchronously infected with A. phagocytophilum were screened with vimentin and SUMO-2/3 antibodies at 8, 12, 16, 24 and 32 h. Uninfected cells served as a control to examine if vimentin is modified by SUMO-2/3 in the absence of infection. Vimentin did not colocalize with SUMO-2/3 in uninfected cells (Figure 3A). However, vimentin and SUMO-2/3 yielded identical patterns around ApVs that strongly colocalized at all time points examined (Figure 3A). The same phenomenon was observed in infected HL-60 cells (Figure 3B). Since we previously determined that AmpA is SUMOylated [14], the localization patterns of vimentin and SUMO-2/3 with AmpA at the ApV were examined in RF/6A cells. SUMO-2/3 and vimentin immunolabeling patterns did not appreciably colocalize with AmpA (Figure 3C). This result and those presented in Figure 3A together suggest that while AmpA is modified by SUMO-2/3 at the ApV membrane [14], vimentin might also be SUMOylated at the ApV. Because SUMO-1 also localizes to ApVs, albeit to a much lesser extent than SUMO-2/3 [14], it was next examined if vimentin colocalizes with SUMO-1 around ApVs. SUMO-1 was detected in infected RF/6A cells throughout the time course and its localization to the AVM was evident by 24 h (Figure 4), as previously observed [14]. However, ApV-associated SUMO-1 and vimentin signals did not colocalize. Thus, vimentin filaments that encage ApVs pronouncedly colocalize with SUMO-2/3 but not SUMO-1 moieties, suggesting that they are polySUMOylated.

### 3.3. Keratin Filaments that Assemble around the ApV Colocalize with SUMO-2/3 Moieties

**Figure 5:**
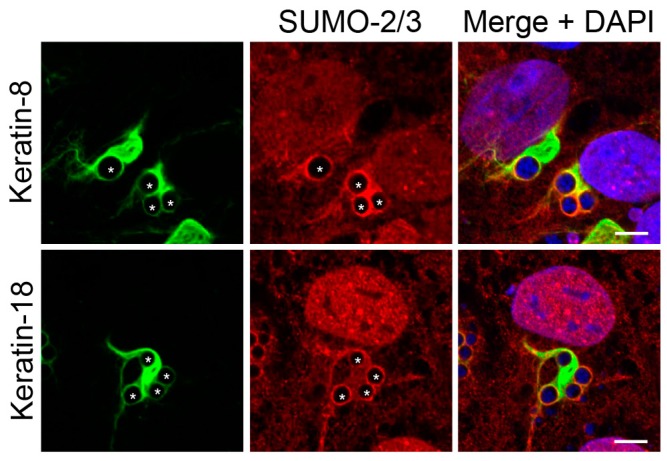
Keratin filaments that assemble around the ApV colocalize with SUMO-2/3 moieties. *A. phagocytophilum* infected RF/6A cells were labeled with SUMO-2/3 and keratin-8 or keratin-18 antibodies and analyzed by LSCM. Asterisks (*) denote ApVs. Host nuclei and bacterial DNA were stained with DAPI (blue). Scale bars, 5 μm. Results in all panels are representative of two experiments with similar results.

Given that keratin is hyperSUMOylated during cellular stress [32], it was next determined if keratin assembled at the ApV also colocalizes with SUMO-2/3 in RF/6A cells. Keratin-8 and keratin-18 wrapped all ApVs, as did SUMO-2/3 moieties (Figure 5). Both keratins exhibited colocalization with SUMO-2/3 around the peripheries of ApVs albeit pronouncedly less than that observed with vimentin and SUMO-2/3 (Figure 3). These data indicate that keratin might also be modified by SUMO-2/3 during *A. phagocytophilum* infection.

### 3.4. Vimentin Expression and the Relative Abundance of Insoluble Vimentin Are Increased in A. phagocytophilum Infected Cells

**Figure 6:**
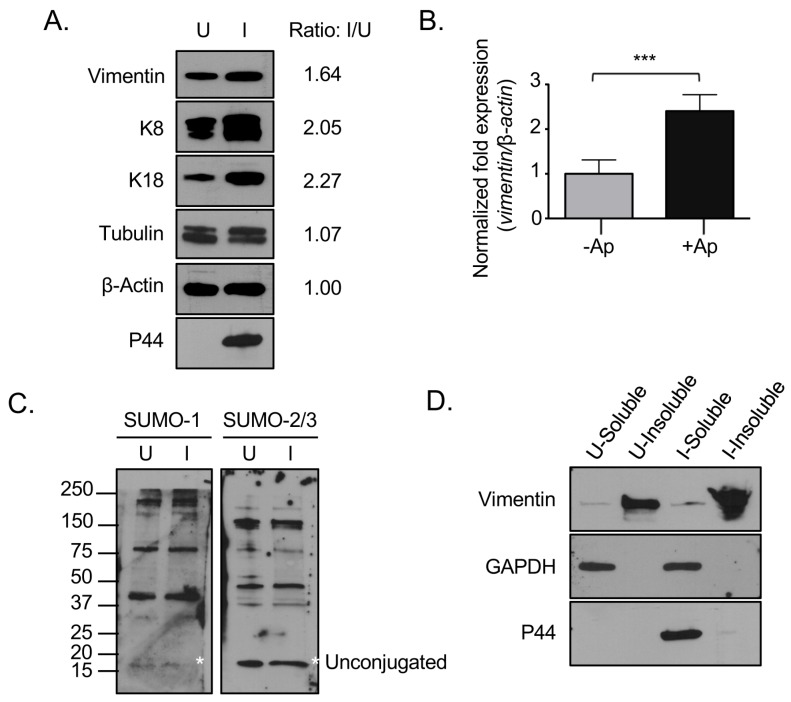
Vimentin expression and the relative abundance of insoluble vimentin are increased in *A. phagocytophilum* infected cells. (**A**) Vimentin and keratin are translationally upregulated in *A. phagocytophilum* infected cells. Lysates of uninfected or *A. phagocytophilum* infected RF/6A cells were analyzed by Western blot with vimentin, keratin-8, keratin-18, tubulin, actin, and P44 antibodies. The ratio of the amount of proteins of interest in infected relative to uninfected cells was determined by densitometry and normalized to actin; (**B**) Vimentin is transcriptionally upregulated in *A. phagocytophilum* infected cells. RNA from uninfected and *A. phagocytophilum* infected RF/6A cells was analyzed by qRT-PCR with vimentin and β-actin primers. Results presented are the means +/− SEM of triplicate samples. Statistically significant differences (*p <* 0.001) are indicated; (**C**) SUMO-1 and SUMO-2/3 levels are not upregulated in infected cells. Lysates of uninfected and *A. phagocytophilum* infected RF/6A cells were analyzed by Western blot with SUMO-1 and SUMO-2/3 antibodies. Asterisks (*) denote the unconjugated SUMO-1 and SUMO-2/3 bands; (**D**) Vimentin remains insoluble in infected cells. Detergent-soluble and detergent-insoluble fractions of uninfected and *A. phagocytophilum* infected RF/6A cells were analyzed by Western blot with vimentin, GAPDH, and P44 antibodies. (**A**,**C**,**D**) U, uninfected; I, infected. Results in all panels are representative of two experiments with similar results.

Given that vimentin immunolabeling was more pronounced in *A. phagocytophilum* infected versus uninfected cells (Figure 1, Figure 2, Figure 3 and Figure 4), we rationalized that vimentin expression might be upregulated during infection. To examine this possibility, lysates of uninfected and infected cells were screened by immunoblot using a vimentin antibody. Keratin and tubulin expression were also evaluated since they also localize to the ApV. The resulting densitometric signals were normalized to β-actin. Antibody against the *A. phagocytophilum* major surface protein, P44 [4], confirmed that the cells were infected. Vimentin, keratin-8, and keratin-18 but not tubulin levels were increased in infected versus uninfected cells (Figure 6A). qRT-PCR analysis established that *A. phagocytophilum* infection results in a 1.5-fold increase in vimentin transcript (Figure 6B). It was next examined if infection results in a corresponding increase in expression of SUMO-1 and SUMO-2/3. Immunoblot analysis of lysates of uninfected and infected RF/6A cells with SUMO-1 and SUMO-2/3 antibodies revealed that unconjugated SUMO-1 and SUMO-2/3 appear to be present in approximately equal amounts in uninfected and infected cells (Figure 6C). The higher molecular weight bands above the unconjugated SUMO moieties correspond to SUMOylated host or bacterial proteins. Notably, there were no detectable differences in the pattern of SUMOylated proteins between uninfected and infected cells.

Unmodified vimentin is largely insoluble, with tissue culture cells maintaining only a small (1%–3%) soluble fraction [45]. MonoSUMOylation of intermediate filaments promotes an increase in the soluble cytosolic pool, while hyperSUMOylation diminishes the soluble cytosolic pool and is associated with the formation of insoluble high molecular weight complexes [29,32]. As vimentin complexes that encage ApVs pronouncedly colocalize with SUMO-2/3, it was next examined if the relative abundance of insoluble vimentin is increased in infected cells. Detergent-soluble and detergent-insoluble fractions were analyzed by Western blot with antibodies against vimentin, host GAPDH, and *A. phagocytophilum* P44, the latter two of which are detergent soluble. While GAPDH and P44 separated into the detergent soluble fraction, vimentin remained largely in the detergent-insoluble fraction in both uninfected and infected cells, which validated the integrity of the fractions (Figure 6D). There was a greater overall abundance of vimentin in the detergent-insoluble fraction of infected cells compared to uninfected cells, but no increase in detergent-soluble vimentin. Taken together, these results indicate that vimentin is upregulated and the relative abundance of insoluble vimentin is increased in *A. phagocytophilum* infected cells.

### 3.5. SUMOylation Is Critical for Vimentin Assembly at the ApV

**Figure 7:**
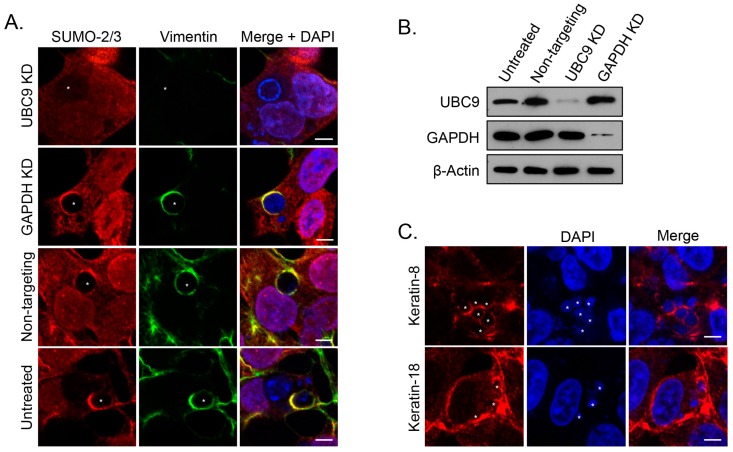
SUMOylation is critical for vimentin assembly at the ApV. (**A**) Knockdown of the SUMO-conjugating enzyme, Ubc9, eliminates vimentin assembly at the ApV. Untreated and HEK-293T cells treated with Ubc9, GAPDH, or non-targeting siRNA were infected with *A. phagocytophilum* and labeled with SUMO-2/3 and vimentin antibodies for LSCM analysis; (**B**) Ubc9 and GAPDH are knocked down in siRNA treated cells. Lysates of untreated and Ubc9, GAPDH, or non-targeting siRNA-treated HEK-293T cells were analyzed by Western blot with Ubc9, GAPDH, and β-actin antibodies. (**A**,**B**) KD, knockdown; (**C**) Keratin-8 and keratin-18 localize to the ApV in *ubc9* knockdown cells. RF/6A cells treated with Ubc9 siRNA were infected with *A. phagocytophilum* and labeled with keratin-8 or keratin-18 antibodies for LSCM; (**A**,**C**) Asterisks (*) denote ApVs. Host nuclei and bacterial DNA were stained with DAPI (blue). Scale bars, 5 μm. Results in all panels are representative of two experiments with similar results.

Given that the vimentin complexes that assemble on the ApV are likely hyperSUMOylated and this modification is critical for promoting the formation of insoluble, high molecular weight complexes [29,32], we posited that SUMOylation is required for the assembly and maintenance of vimentin at the ApV. Accordingly, vimentin association with ApVs was assessed in HEK293T cells that had been treated with siRNA targeting the ubiquitin conjugating enzyme, Ubc9. HEK-293T cells were chosen because they are susceptible to *A. phagocytophilum* infection and have a high transfection efficiency (≥75%) relative to RF/6A and HL-60 cells (<20%) [14,15,16,46]. As controls, the cells were also treated with GAPDH-targeting and non-targeting siRNA. Knockdown of Ubc9 and GAPDH was verified by Western blot (Figure 7B). Untreated and siRNA treated cells were infected with *A. phagocytophilum*, labeled with vimentin and SUMO-2/3 antibodies, and examined by confocal microscopy. In Ubc9 siRNA treated cells only, the association of vimentin with the ApV was completely lost (Figure 7A). Notably, however, and consistent with our previous reports [14], the *A. phagocytophilum* load was unaffected by Ubc9 knockdown (data not shown). To determine if intermediate filaments other than vimentin might still structurally support the ApV, we probed Ubc9 siRNA treated infected cells with antibodies to keratin-8 and keratin-18. Filaments comprised of both keratin subunits pronouncedly wrapped ApVs in both Ubc9 knockdown and control cells (Figure 7C). Thus, consistent with our colocalization data presented in Figure 1, Figure 2, Figure 3, Figure 4 and Figure 5, SUMOylation is important for vimentin but not keratin assembly at the ApV.

### 3.6. Active Bacterial Protein Synthesis Is Not Necessary for Vimentin Assembly and SUMO-2/3 Localization at the ApV

**Figure 8:**
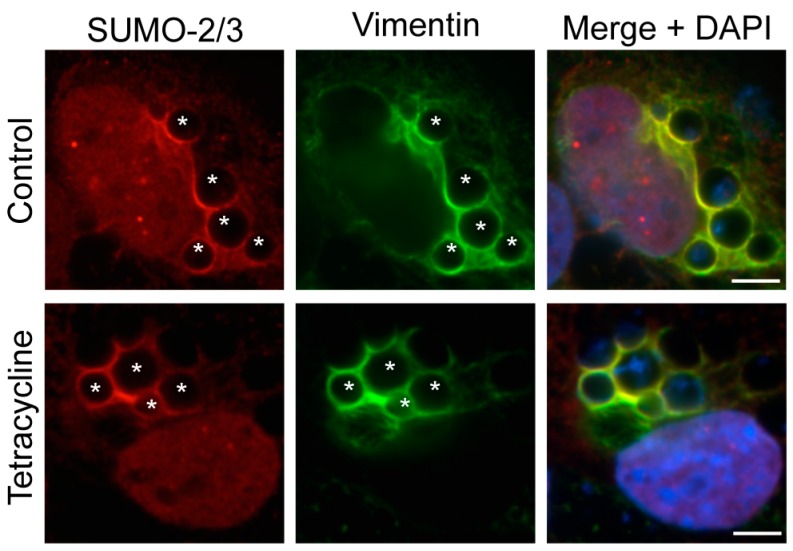
Active bacterial protein synthesis is not necessary for the assembly of vimentin and SUMO-2/3 moieties at the ApV. *A. phagocytophilum* infected RF/6A cells were treated with tetracycline or vehicle control. At 5 h, tetracycline was removed to restore bacterial protein synthesis. Cells were collected at 0, 1, 2, and 4 h post-washing and labeled with SUMO-2/3 and vimentin antibodies. The images shown were taken at 0 h post-washing, but are representative of what was observed at all post-wash time points. Host nuclei and bacterial DNA were stained with DAPI. Asterisks (*) denote representative ApVs. Scale bars, 5 μm. Results in all panels are representative of two experiments with similar results.

It was next determined if nascent bacterial protein synthesis is necessary for maintaining vimentin assembly on and/or SUMO-2/3 localization to the ApV. RF/6A cells that had been infected for 24 h and therefore harbored ApVs to which vimentin and SUMO-2/3 moieties had localized, were treated with tetracycline, which exerts its bacteriostatic effect by inhibiting bacterial protein synthesis. After 5 h, the cells were washed and the media replaced to allow for bacterial protein synthesis to resume. At 0, 1, 2, and 4 h post-washing, the cells were examined by LSCM. Vimentin assembly and SUMO-2/3 localization to the ApV were unaffected at all time points (Figure 8 and data not shown), indicating that once vimentin and SUMO-2/3 moieties have assembled on the ApV, active bacterial protein synthesis is not necessary to maintain them at this locale.

### 3.7. Pharmacologic Inhibition Soluble Vimentin Reduces the A. phagocytophilum Load

**Figure 9:**
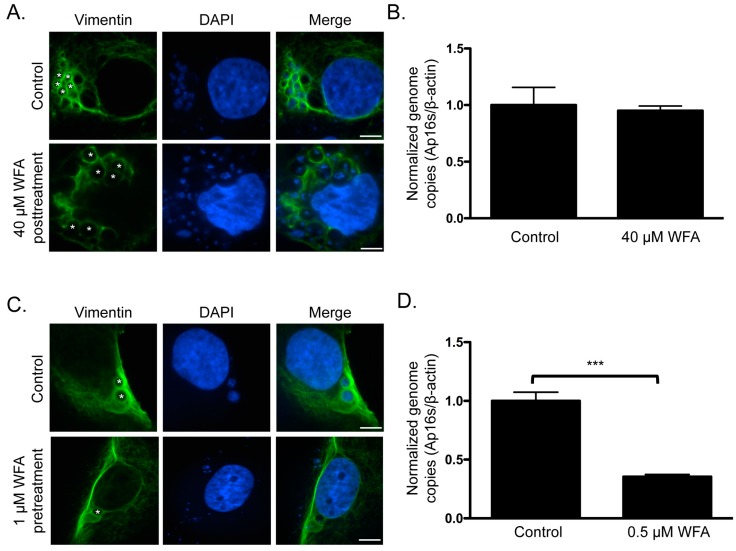
Pharmacologic inhibition of soluble vimentin reduces the *A. phagocytophilum* load. (**A**,**B**) Treatment of *A. phagocytophilum* infected cells with WFA does not reduce vimentin assembly around the ApV or alter the bacterial DNA load. *A. phagocytophilum* infected RF/6A cells were treated with 40 μM WFA for 3 h. Cells were screened with vimentin antibody, stained with DAPI, and analyzed using LSCM (**A**) or subjected to QPCR analysis using primers targeting *A. phagocytophilum* 16S rDNA and β-actin (**B**); (**C**,**D**) Treatment with WFA prior to and during *A. phagocytophilum* infection inhibits vimentin assembly around the ApV and reduces the bacterial DNA load. RF/6A cells were pretreated with 0.5 μM or 1 μM WFA for 2 h and then infected with *A. phagocytophilum* in the continued presence of WFA. At 24 h, the cells were analyzed by LSCM (**C**) and QPCR (**D**). Statistically significant values (*** *p* < 0.001) are indicated. Results in (**A**,**C**) and (**B**,**D**) are representative of three and four experiments with similar results, respectively. (**A**,**C**) Asterisks (*) denote representative ApVs. Scale bars, 5 μm.

Given the pronounced localization of vimentin at the ApV that is maintained throughout infection, we rationalized that this might be important for optimal *A. phagocytophilum* growth. It was examined if treatment with WFA, which binds to and inhibits soluble vimentin [47], reduces its association with the ApV and, if so, whether this affects the bacterial load. First, *A. phagocytophilum* infected RF/6A cells were treated with WFA at concentrations ranging from 100 nM to 40 μM for 3 h. WFA failed to inhibit vimentin assembly around ApVs and had no effect on the bacterial DNA load even at the highest concentration examined (Figure 9A,B). The drug was toxic to host cells at concentrations above 40 μM. Thus, once vimentin polymerization around the ApV has occurred, its association with the ApV is stable. Furthermore, it indirectly indicated that WFA exerted no off-target effect on bacterial growth or viability. Given that the vimentin assembled around ApVs was likely in the insoluble form and thus would be insensitive to WFA, we examined the effects of treating host cells with the inhibitor prior to and during infection. RF/6A cells were treated with 0.5 or 1 μM WFA for 2 h followed by incubation with *A. phagocytophilum* organisms and continued cultivation in the presence of WFA for 24 h. Because of the duration of WFA treatment, such low concentrations were essential to minimize toxicity. Relative to vehicle control treated infected cells and consistent with a previous report [47], vimentin filaments yielded a perinuclear concentration in WFA treated infected cells (Figure 9C). For those ApVs that did form, vimentin localization was less pronounced and fewer DAPI-stained bacteria were detected within their lumen. In addition, the *A. phagocytophilum* DNA load was reduced by more than 50% (Figure 9D). Thus, inhibition of soluble vimentin prior to and during *A. phagocytophilum* infection inhibits optimal ApV formation and bacterial growth.

## 4. Discussion

*A. phagocytophilum* is an obligate intracellular pathogen that replicates in a membrane-bound vacuole that interfaces with different host cells organelles and vesicular traffic. As organelles and vesicles are anchored, stabilized, and trafficked by cytoskeletal components, we hypothesized that the ApV might associate with components of the cytoskeleton. Here, we demonstrate that the ApV is surrounded by the intermediate filaments vimentin and keratin and also microtubules. Vimentin assembly around the ApV early during infection is important for its optimal formation and bacterial intracellular growth, a finding that is in agreement with a report that vimentin assembly at the ApV mediated by the effector, AptA, activates Erk/MAPK to promote *A. phagocytophilum* survival. Pretreatment of host cells with 0.5 or 1.0 uM WFA blocked vimentin assembly at the ApV and reduced bacterial load. As with any pharmacologic inhibitor, off target effects of WFA cannot be absolutely ruled out. However, given that 40 μM WFA had no effect on the *A. phagocytophilum* DNA load when administered post infection, the observed reduction in the bacterial load with pretreatment of WFA is most likely a specific consequence of the drug’s inhibition of soluble vimentin. Thus, once vimentin has assembled on the ApV, its association is very stable, as it is no longer sensitive to WFA and does not require nascent bacterial protein synthesis to be maintained.

While much is known about how many intracellular bacteria manipulate cytoskeletal functions at the plasma membrane to promote uptake into host cells [25], comparatively very little is known about how they interact with the cytoskeleton once inside the host cell. Of what information exists, most derives from studies of the *Salmonella*-containing vacuole (SCV) and the *Chlamydia trachomatis* inclusion. The SCV has been found to associate with microtubules, the actin-based motor myosin II, and the intermediate filaments vimentin, keratin-8, and keratin-18 [25]. Association of myosin II and intermediate filaments promotes stability of the SCV and its juxtanuclear position in the host cell [48,49], while microtubules promote SCV movement late in infection to the cell periphery for dissemination [50]. Similarly, *C. trachomatis* induces reorganization of the intermediate filaments vimentin, keratin-8, and keratin-18 as well as filamentous actin at the inclusion for vacuole stability [20]. Disruption of actin or the intermediate filaments leads to a loss of inclusion integrity, dispersal of bacteria into the host cytosol and detection by immune surveillance mechanisms. Microtubules were not detected at the chlamydial inclusion. The researchers hypothesized that the microtubules might be displaced by the inclusion, which would obscure their visualization [20]. Likewise, we do not believe that the present study precludes the possibility of actin association with the ApV. It is important to note, however, that association of cytoskeletal elements, including actin, with intracellular bacteria or pathogen-occupied vacuoles is not always advantageous. Thus, pathogens may have evolved to avoid certain interactions. Polymerization of actin at the SCV in macrophages, for example, promotes inflammasome activation that impedes bacterial proliferation [51]. Additionally, a proportion of cytosolic *Shigella* and *Mycobacterium marinum* are wrapped in septin filaments, referred to as “the fourth component of the cytoskeleton” [52], which culminates in their destruction in autophagosomes [25].

In addition to modulating Erk/MAPK signaling, we hypothesize that, similar to *C. trachomatis* [20], the structural scaffold of vimentin surrounding the ApV provides vacuole stability. Unfortunately, utilizing knockdown of vimentin [17] and Ubc9 to examine this phenomenon is confounded by the presence of cytokeratins at the ApV, which conceivably also stabilize the vacuole and explain the lack of reduced bacterial numbers after knockdown. Remarkably, vimentin also plays a role in impeding bacterial killing by professional phagocytes [53]. Through its interaction with the p47^phox^ active subunit of NADPH oxidase, vimentin suppresses the production of reactive oxygen species and the respiratory burst. Though *A. phagocytophilum* is known to employ multiple mechanisms to inhibit the respiratory burst [54,55,56,57,58,59,60,61,62,63], we cannot discount the possibility that assembly of vimentin at the ApV may also contribute. Microtubules also assemble at the ApV, albeit considerably less than vimentin or the cytokeratins. As microtubules are molecular motors that allow organelle and vesicle movement throughout the cell, we hypothesize that they contribute to the movement of the ApV to the perinuclear region of the host cell [15], similar to *Salmonella* infection, and may also help facilitate recruitment of autophagosomes as well as *trans*-Golgi- and ER-derived vesicles to the ApV [15,16,18].

Vimentin is upregulated, perfectly colocalizes with SUMO-2/3 but not SUMO-1, and is detergent-insoluble in *A. phagocytophilum*-infected cells. As SUMO-2/3 moieties are conjugated as polymers and SUMO-1 as monomers [29], these results indicate that vimentin is likely polySUMOylated in infected cells, a property that regulates filament formation and retains vimentin in an insoluble, high molecular mass complex [29,32,64]. Unfortunately, technical issues owing to this transient nature of SUMOylation [65] and the extreme insolubility of vimentin [66], which requires harsh detergents to solubilize it from the cell that then complicate mass spectrometry analysis and immunoprecipitation studies prevented biochemical confirmation of this modification. However, these data, the WFA posttreatment, which had no effect on vimentin assembly, and the knockdown of Ubc9, which abrogated vimentin assembly at the ApV, strongly suggest that vimentin that assembles around the ApV is polySUMOylated. Keratin-8 and keratin-18 that localize to the ApV might also be SUMOylated. Intermediate filaments, keratins specifically, have been shown to undergo polymeric SUMOylation by SUMO-2/3 (hyperSUMOylation) and dramatic filament reorganization as a deleterious response to oxidative and apoptotic stress [29]. In contrast, other studies have shown that SUMOylation of intermediate filaments is important for their assembly and function [27,64,67]. Given that vimentin is SUMOylated during *A. phagocytophilum* infection and that both vimentin and SUMOylation are important for *A. phagocytophilum* growth [14,17], vimentin SUMOylation is likely not a host response induced by infection but rather is a pro-microbial, bacterial-driven process. Many bacterial pathogens, including *Listeria monocytogenes*, *Yersinia* spp., and *Xanthomonas campestris* pv. *vesicatoria*, negatively modulate SUMOylation [68,69,70]. *A. phagocytophilum* and the closely phylogenetically related intracellular bacterium, *Ehrlichia chaffeensis*, are the only two pathogens identified thus far that promote and benefit from SUMOylation [14,71]. Whether or not *A. phagocytophilum* directly influences the SUMOylation of vimentin and other host proteins will be an important future point of study.

## 5. Conclusions

This study demonstrates that vimentin, keratin, and microtubules assemble on the ApV, vimentin is likely hyperSUMOylated during *A. phagocytophilum* infection, and this modification is important for its assembly/retention at the ApV. Vimentin assembly at the ApV is important for its optimal development and *A. phagocytophilum* intracellular growth. Once vimentin has been recruited and polymerized into its insoluble form around the ApV, its association with the pathogen-occupied is very stable, WFA-insensitive, and nascent bacterial protein synthesis is unnecessary for maintaining this association. Given that vimentin and its assembly at the ApV are pro-microbial in *A. phagocytophilum* infection and in other bacterial infections [17,25,53], this study highlights a potentially conserved mechanism by which intracellular pathogens exploit intermediate filaments for survival.

## Abbreviations

The following abbreviations are used in this manuscript:

ApV	*Anaplasma phagocytophilum*-occupied vacuole
LSCM	Laser-scanning confocal microscopy
WFA	Withaferin A
SCV	*Salmonella*-containing vacuole
Erk1/2	Extracellular signal-related kinases 1 and 2
MAPK	Mitogen-associated protein kinases
